# Expression of miR‐18a and miR‐34c in circulating monocytes associated with vulnerability to type 2 diabetes mellitus and insulin resistance

**DOI:** 10.1111/jcmm.13240

**Published:** 2017-06-29

**Authors:** Si‐Si Wang, Yong‐Qiang Li, Ying‐Zhi Liang, Jing Dong, Yan He, Ling Zhang, Yu‐Xiang Yan

**Affiliations:** ^1^ Department of Epidemiology and Biostatistics School of Public Health Capital Medical University Beijing China; ^2^ Municipal Key Laboratory of Clinical Epidemiology Beijing China; ^3^ Department of Nephrology Institute of Nephrology and Urology The Third Affiliated Hospital of Southern Medical University Guangzhou China; ^4^ Health Medical Examination Center Xuanwu Hospital Capital Medical University Beijing China

**Keywords:** MicroRNA, Stress, Type 2 diabetes mellitus, Insulin resistance, Biomarker

## Abstract

Chronic stress may facilitate the development of metabolic disorders including insulin resistance (IR) and type 2 diabetes mellitus (T2DM). MiR‐18a and miR‐34c modulate central cell responsiveness to stress by targeting glucocorticoid receptor (GR) and corticotropin‐releasing factor receptor type 1 (CRFR1) mRNA, which are important regulators of the hypothalamus–pituitary–adrenal (HPA) axis. This study explored the relationship between T2DM/IR and expression of miR‐18a and miR‐34c in peripheral blood mononuclear cells (PBMCs) in an occupational sample. Three groups of study subjects were involved, including T2DM patients, impaired fasting glucose (IFG) individuals and healthy controls. The degree of IR was determined using the homoeostasis model assessment of insulin resistance (HOMA‐IR). The expression of miR‐18a and miR‐34c in PBMCs was evaluated by quantitative reverse transcription polymerase chain reaction (qRT‐PCR). Expression levels of miR‐18a and miR‐34c were significantly correlated with cortisol, corticotropin‐releasing factor (CRF) and interleukin 6 (IL‐6) (*P* < 0.05). The increased levels of miR‐18a were associated with risk of T2DM (adjusted OR = 1.48, 95% CI: 1.25–1.75, *P* < 0.001) and IFG (adjusted OR = 1.33, 95% CI: 1.09–1.63, *P* = 0.005). By contrast, the decreased levels of miR‐34c were associated with risk of T2DM (adjusted OR = 0.81, 95% CI: 0.75–0.88, *P* < 0.001) and IFG (adjusted OR = 0.87, 95% CI: 0.81–0.94, *P* < 0.001). After adjusting for potential confounders, miR‐18a and miR‐34c were independent positive and negative predictors of HOMA‐IR, respectively (*P* < 0.001). The miRNA panel with the two miRNAs demonstrated high accuracy in the diagnosis of T2DM (AUC = 0.851, 95% CI: 0.786–0.800, *P* < 0.001). MiR‐18a and miR‐34c in PBMCs may be important marker of stress reaction and may play a role in vulnerability to T2DM as well as IR.

## Introduction

Globally, T2DM is one of the most prevalent chronic illnesses. Managing T2DM is a major challenge now affecting the lives of around 300 million people. This phenomenon is particularly evident in inland China, which has the prevalence of 11.6% for diabetes in adults 1. In addition to genetic causes, environmental influences such as chronic stress, behavioural disturbances and dietary deficiency have now emerged as contributors to the development of T2DM. Psychological stress can affect health through complex interactions among neuroendocrine responses and energy homoeostasis 2, 3. One of the major neuroendocrine systems responding to psychological stress is the HPA axis, with cortisol secretion as its final hormonal effector. Our previous study showed that chronic stress is a dependent risk factor of IR 4. Cortisol was significantly positively correlated with glucose, HOMA‐IR and waist circumference (WC) in males and females (*P* < 0.05).

MicroRNAs (miRNAs) are a class of 22 nucleotide short noncoding RNAs that play key roles in regulation of cellular processes in response to changes in environment. Emerging data suggest that stress conditions can alter the biogenesis of miRNAs, the activities of miRNA‐protein complexes and the expression of mRNA targets 5, 6. Uchida and colleagues identified that miR‐18a inhibited translation of GR mRNA in cultured neuronal cells and that high expression of miR‐18a was present in Fischer 344 (F344) rats in the paraventricular nucleus (PVN) 7. The function of miR‐34c in regulating stress responses was investigated by Haramati *et al*. who showed that miR‐34c elicits its effect on the amygdala by targeting an evolutionarily conserved region in the 3′ UTR of CRFR1 mRNA 8. GR and CRFR1 are important regulators of stress‐induced activation of the HPA axis. Most effects of cortisol and CRF are mediated by the GR and CRFR1. Investigation in an occupational population showed that chronic stress increases plasma cortisol, which subsequently impacts GR expression in PBMCs, including decreased mRNA expression of GRα and increased GRβ/GRα mRNA ratio 9. We predict that miR‐18a and miR‐34c may involve in a variety of systemic stress responses and contribute to vulnerability of disease.

To our knowledge, few studies have investigated expression of stress‐related circulating miRNAs in T2DM population. It has been suggested that gene expression signature in PBMCs may provide an indicator of gene activation changes as differential response to stress in humans 10. In this study, we analysed association between T2DM and expression of miR‐18a and miR‐34c in PBMCs, which may unveil new target for the prevention and treatment of stress‐related disorders including T2DM.

## Materials and methods

### Subjects

This study was conducted among workers who took annual physical examination for at least three consecutive years at the health examination centre of Beijing Xuanwu Hospital, Capital Medical University during March to October 2015. A total of 117 newly diagnosed T2DM cases aged from 30 to 65 years old were recruited. 105 health individuals with normal glucose (<6.1 mmol/l) were selected as frequency matched controls according to gender and age (±3 years). Another 74 individuals with IFG were also recruited. The diagnosis of T2DM and IFG was in accordance with the World Health Organization (WHO) criteria 11. T2DM = Fasting glucose (FG) levels ≥7.0 mmol/l or 2 hrs glucose levels ≥11.1 mmol/l in an oral glucose. IFG = FG ≥6.1 mmol/l and <7.0 mmol/l.

Patients with a past history of T2DM and using antidiabetic drugs according to the medical records were excluded from the study. Patients with acute or chronic infectious or immunological diseases, obvious liver and kidney dysfunction, severe heart diseases, hypertension, pregnancy, mental illness or drug abuse, gastrointestinal diseases (such as chronic gastrointestinal disorders, diarrhoea, biliary tract infection and enteritis), serious diseases of the blood or the endocrine systems, type I DM or other particular types of DM were also excluded.

A structured questionnaire was used to collect information on demographic data, environmental exposure and medical histories 12. This study was approved by the university ethical committee, and informed consent was obtained from each participant.

### Anthropometric measurement

Anthropometric parameters including weight, height, WC and blood pressure were obtained using standard measurement described previously 4. Body mass index (BMI) was calculated by dividing the weight (kg) by the squared value of height in metres.

### Blood samples collection and RNA extraction

Following an overnight fast, 5 ml venous blood sample from each subject was collected using EDTA anticoagulant tubes and processed within an hour. Three millilitre sample was immediately centrifuged to retrieve plasma. PBMCs were isolated from 2 ml whole blood by Ficoll‐Hypaque density gradient centrifugation. Immediately after, total RNA was extracted from PBMCs by standard protocol of Trizol reagent (Invitrogen, New York, USA). The yield of RNA was determined using a NanoDrop 2000 spectrophotometer (Thermo Scientific, Waltham, MA, USA), and the integrity was evaluated using agarose gel electrophoresis stained with ethidium bromide. All RNA used had A260/280 ratio >1.8, and electrophoresis showed integrity is acceptable. The plasma and RNA were then stored at −80°C until assayed.

### Biochemical analysis

Fasting plasma glucose (FPG) total cholesterol (TC), triglycerides (TG) and high‐density lipoprotein cholesterol (HDLC) were measured using standard laboratory methods (Hitachi autoanalyzer 7060; Hitachi, Tokyo, Japan). Low‐density lipoprotein cholesterol (LDLC) was calculated using the Friedewald formula. Glycated haemoglobin (HbAlc) was estimated by high‐pressure liquid chromatography method (Tosoh Corporation, Tokyo, Japan).

Plasma cortisol and insulin were measured by commercial radioimmunoassays using with a γ counter (XH‐6020; North Institute of Bio‐Tech, Beijing, China). Plasma CRF and IL‐6 concentrations were evaluated by enzyme‐linked immunosorbent assay using microplate reader (STAT FAX 2100; Awareness, Palm City, FL, USA). The intra‐assay and inter‐assay coefficient of variation were <5.5% and <10.0% for these assays, respectively. The degree of IR was determined using the HOMA‐IR. HOMA‐IR was calculated using the following formula: [fasting insulin (μIU/ml) × fasting glucose (mmol/l)]/22.5.

### Quantitative real‐time PCR

Quantification was performed with a two‐step reaction process: reverse transcription (RT) and quantitative real‐time PCR (qPCR). Each RT reaction consisted of 1 μg RNA, 4 μl of miScript HiSpec Buffer, 2 μl of Nucleics Mix and 2 μl of miScript Reverse Transcriptase Mix (Qiagen, Hamburg, Germany), in a total volume of 20 μl. Reactions were performed in a GeneAmp^®^ PCR System 9700 (Applied Biosystems, Foster City, USA) for 60 min. at 37°C, followed by heat inactivation of RT for 5 min. at 95°C. The 20 μl RT reaction mix was then diluted × 5 in nuclease‐free water and held at −20°C. qPCR was performed using LightCycler^®^ 480 Instrument II (Roche, Rotkreuz, Switzerland) with 10 μl PCR reaction mixture that included 1 μl of cDNA, 5 μl of 2 × LightCycler^®^ 480 SYBR Green I Master (Roche), 0.2 μl of universal primer (Qiagen), 0.2 μl of miRNA‐specific primer and 3.6 μl of nuclease‐free water. Reactions were incubated in a 384‐well optical plate at 95°C for 10 min., followed by 40 cycles of 95°C for 10 sec., 60°C for 30 sec. The specific generation of expected PCR product was confirmed by automated melting curve analysis. All samples were performed in triplicate.

Two housekeeping genes including miR‐16 and RNU6B were used. As RNU6B was quite consistent among the three groups, it was chosen as the reference miRNA for data analyse. Triplicate values of each sample were normalized to RNU6B. The expression levels of miRNAs were calculated using the 2^−ΔCt^ method [ΔCt = mean Ct (miRNA of interest)‐mean Ct (RNU6B)].

The miRNA‐specific primer sequences were designed in the laboratory and synthesized by Generay Biotech (Generay, PRC) based on the miRNA sequences obtained from the miRBase database (Release 20.0) as follows: miR‐18a ‐forward: 5′‐taaggtgcatctagtgcagatag‐3′; miR‐34c ‐forward: 5′‐aggcagtgtagttagctgattgc‐3′, u6‐forward: 5′‐CAAGGATGACACGCAAATTCG‐3′.

### Validation of the miRNA quantification

The results of miRNA quantification were further validated in a 20% of repeated samples using the following TaqMan MicroRNA Assays (Applied Biosystems): miR‐18a (ID: 002422), miR‐34c (ID: 000428), RNU6B (ID: 001093). RT and qPCR were performed according to instructions of the manufacturer. Briefly, 10 ng RNA was reversely transcribed into cDNA using TaqMan MicroRNA Reverse Transcription Kit (Applied Biosystems). qPCR was then performed using TaqMan Universal PCR Master Mix No AmpErase UNG (Applied Biosystems) in a final volume of 10 μl with the ABI 9700 PCR system. Correlations between both methods indicated that the results of miRNA quantification were reliable (*r*_miR‐18_ = 0.957 and *r*_miR‐34c_ = 0.936, *P* < 0.001).

### Statistical analysis

Normality of data distribution was assessed using the Kolmogorov–Smirnov test. One‑way analysis of variance (anova) and chi‐square test were used to compare differences of demographic and clinical parameters. Least significant difference (LSD) analysis test was used for multiple comparisons. Spearman's correlation coefficient was used to test the correlation between miRNA markers and Cortisol, CRF and other clinical variables. The odds ratios (ORs) and their 95% confidence intervals (CIs) were calculated to assess the risk of miR‐18a and miR‐34c contributed to the presence of T2DM and IFG using both univariate and multivariate logistic regression models with or without adjustment for covariates. A stepwise multiple regression analysis was performed to identify the predictors of HOMA‐IR with each miRNA expression level itself was used as a variable factor. Receiver operating characteristic (ROC) analysis was used to assess the biomarker potential of each miRNA for T2DM, and area under the curve (AUC) was used as diagnostic index. The diagnostic performance of the miRNA panel was further evaluated using the predicted probability of being diagnosed with T2DM as a surrogate marker to construct ROC curve. A *P* value of less than 0.05 was considered statistical significance. The reported *P* values were two‐tailed in all calculations. All statistical analyses were performed using SPSS 20.0 (IBM SPSS, Inc., Chicago, IL, USA).

## Results

### Basic characteristics of the study subjects

The demographic and clinical characteristics of the study participants were presented in Table 1. There was no significant difference in the distribution of age, gender, smoking, alcohol use and TC (*P* > 0.05). Compared with those controls, subjects with IFG and T2DM were more likely to be physical inactivity (*P* < 0.05). Most of the demographic parameters, including BMI, WC, SBP, DBP, TG and HDLC, were significantly higher in IFG and T2DM group than those in control group (*P* < 0.05). anova revealed significant differences for FPG, HbA1c, insulin and HOMA‐IR among the three groups (*P* < 0.001).

**Table 1:** Demographic and clinical characteristics of study subjects

Variable	T2DM (*n* = 117)	IFG (*n* = 74)	Control (*n* = 105)	*P*
Age(year)	51.68 ± 8.77	51.09 ± 7.48	49.26 ± 9.09	0.101
Gender(male/female)	68/49	42/32	58/47	0.911^a^
BMI (kg/m^2^)	27.44 ± 3.08^b^	26.70 ± 3.25^b^	24.18 ± 2.86	<0.001
WC (cm)	90.50 ± 8.92^b^	88.58 ± 9.63^b^	82.42 ± 9.63	<0.001
SBP (mmHg)	133.89 ± 16.23^b^	133.60 ± 18.84^b^	123.14 ± 17.12	<0.001
DBP (mmHg)	83.39 ± 11.36^b^	82.85 ± 11.91^b^	76.34 ± 9.80	<0.001
FPG (mmol/l)	9.51 ± 2.92^b^^,^ c	6.54 ± 0.24^b^	5.03 ± 0.46	<0.001
TC (mmol/l)	5.21 ± 1.53	5.01 ± 1.14^b^	4.93 ± 0.96	0.225
TG (mmol/l)	2.61 ± 3.14^b^	2.40 ± 3.71^b^	1.35 ± 0.83	0.002
HDLC (mmol/l)	1.39 ± 0.36^b^	1.35 ± 0.33^b^	1.56 ± 0.45	<0.001
LDLC (mmol/l)	3.22 ± 1.02^b^^,^ c	2.90 ± 0.84	2.86 ± 0.84	0.007
HbA1c (%)	7.51 ± 1.42^b^^,^ c	5.75 ± 0.59^b^	5.19 ± 0.42	<0.001
Insulin (uIU/ml)	15.06 ± 2.97^b^^,^ c	13.54 ± 3.14^b^	11.77 ± 2.99	<0.001
HOMA‐IR	6.41 ± 2.46^b^^,^ c	3.94 ± 0.93^b^	2.64 ± 0.73	<0.001
Smoking (*n*, %)	21, 17.95	12, 16.22	10, 9.52	0.184^a^
Alcohol use (*n*, %)	14, 11.96	13, 17.57	7, 6.67	0.077^a^
Physical activity (*n*, %)	73, 62.39^b^	50, 67.57^b^	82, 78.10	0.038^a^

BMI: body mass index, WC: waist circumference, SBP: systolic blood pressure, DBP: diastolic blood pressure, FPG: fast plasma glucose, TC: total cholesterol, TG: triglyceride, HDLC: high‐density lipoprotein cholesterol, LDLC: low‐density lipoprotein cholesterol, HOMA‐IR: homoeostasis model assessment of insulin.

a χ^2^ value.

b Significantly different from control group (*P* < 0.05).

c Significantly different from IFG group (*P* < 0.05).

### Comparison of miRNA levels and stress hormone among control, IFG and T2DM groups

**Figure 1:**
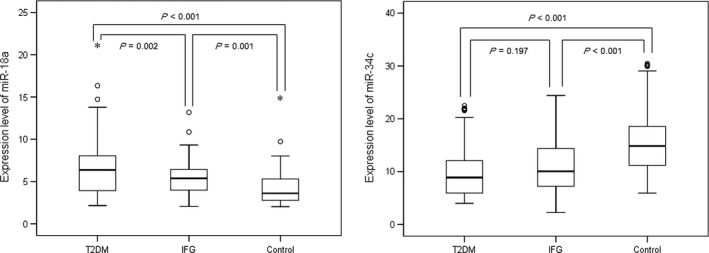
Comparison of microRNA expression in PBMCs among T2DM, IFG and control groups.

The expression levels of miR‐18a and miR‐34c in the PBMCs and plasma levels of stress hormones including cortisol, CRF and IL‐6 of the three groups were listed in Table 2. The expression levels of miR‐18a in T2DM patients were significantly higher than that in IFG and control individuals (*P* = 0.002 and *P* < 0.001, respectively). IFG subjects had significantly higher levels of miR‐18a, as compared to control individuals (*P* = 0.001) (Fig. 1). The expression levels of miR‐34c in subjects with IFG and T2DM were significantly lower than that in controls (*P* < 0.001). However, no significant difference was observed between IFG and T2DM individuals (*P* = 0.197) (Fig. 1).

**Table 2:** MiRNA and hormonal characteristics in the three compared groups

Variable	T2DM (*n* = 117)	IFG (*n* = 74)	Control (*n* = 105)	*P*
miR‐18a	6.63 ± 3.19^b^^,^ c	5.43 ± 2.02^b^	4.19 ± 1.96	<0.001^a^
miR‐34c	9.95 ± 4.92^b^	11.02 ± 5.44^b^	15.98 ± 6.25	<0.001^a^
Cortisol (ng/ml)	220.52 ± 36.64^b^^,^ c	202.73 ± 35.77^b^	177.60 ± 35.84	<0.001
CRF (ng/ml)	5.38 ± 0.66^b^	5.36 ± 0.56^b^	5.03 ± 0.46	<0.001
IL‐6 (pg/ml)	133.42 ± 18.96^b^^,^ c	127.17 ± 19.12	123.04 ± 21.67	0.001

CRF: corticotropin‐releasing factor, IL‐6: interleukin 6, HOMA‐IR: homoeostasis model assessment of insulin.

a Skewed distributed and analysed by log‐transformed values.

b Significantly different from control group (*P* < 0.05).

c Significantly different from IFG group (*P* < 0.05).

With respect to stress hormone, significant differences were also seen among the three studied groups (*P* < 0.05). Plasma cortisol levels were significantly higher in T2DM patients than that in IFG individuals (*P* = 0.001), while the latter group showed higher levels of cortisol than the control group (*P* < 0.001). Plasma CRF levels were significantly higher in IFG and T2DM groups than those in control group (*P* < 0.001). Plasma IL‐6 levels were significantly higher in T2DM group than those in IFG and control groups (*P* = 0.036 and *P* < 0.001).

### Risk of miRNA expression contributed to the presence of T2DM and IFG

Univariate logistic regression revealed that expression levels of miR‐18a and miR‐34c were significantly associated with the presence of T2DM and IFG (*P <* 0.05) (Table 3). These associations were also confirmed in multivariate logistic regression analysis after adjustment for age, gender, smoking, drinking and physical activity, additionally for WC, and further for blood lipids and blood pressure. With a unit increase of miRNA‐18a level, there was 1.48 (95% CI: 1.25–1.75, *P* < 0.001) times of greater risk of T2DM and 1.33 (95% CI: 1.09–1.63, *P* = 0.005) times of greater risk of IFG. By contrast, each unit increase of miR‐34c level was associated with a decreased risk of T2DM (adjusted OR = 0.81, 95% CI: 0.75–0.88, *P* < 0.001) and IFG (adjusted OR = 0.87, 95% CI: 0.81–0.94, *P* < 0.001). These results indicate that miR‐18a is an independent risk factor and miR‐34c is an independent protective factor for T2DM, respectively.

**Table 3:** Univariate and multiple logistic regression analysis for the risk of T2DM and IFG

Models	T2DM		IFG	
	OR (95% CI)	*P* value	OR^a^ (95% CI)	*P* value
miR‐18a				
Univariate model	1.52 (1.32, 1.75)	<0.001	1.38 (1.17, 1.64)	<0.001
Multivariate model 1^a^	1.50 (1.30, 1.73)	<0.001	1.36 (1.14, 1.63)	0.001
Multivariate model 2^b^	1.43 (1.23, 1.66)	<0.001	1.32 (1.09, 1.59)	0.004
Multivariate model 3^c^	1.48 (1.25, 1.75)	<0.001	1.33 (1.09, 1.63)	0.005
miR‐34c				
Univariate model	0.82 (0.77, 0.87)	<0.001	0.89 (0.81, 0.92)	<0.001
Multivariate model 1^a^	0.82 (0.77, 0.87)	<0.001	0.85 (0.79, 0.91)	<0.001
Multivariate model 2^b^	0.83 (0.77, 0.88)	<0.001	0.86 (0.80, 0.92)	<0.001
Multivariate model 3^c^	0.81 (0.75, 0.88)	<0.001	0.87 (0.81, 0.94)	<0.001

OR: odds ratio, CI: confidence interval.

a Adjusted for age, gender, smoking, drinking and physical activity.

b Further adjusted for WC based on model 1.

c Further adjusted for TC, TG, HDLC, LDLC, SBP and DBP based on model 2.

### The correlation of miRNA expression with stress hormones

The spearman correlation analysis showed that plasma stress hormones were significantly associated with miR‐18a and miR‐34c expression in the study subjects (Table 4). MiR‐18a was positively correlated with cortisol (*P* < 0.001), CRF (*P* < 0.001) and IL‐6 (*P* = 0.002), while miR‐34c was negatively correlated with cortisol (*P* < 0.001), CRF (*P* < 0.001) and IL‐6 (*P* = 0.041). Significant positive correlation between miR‐18a and cortisol (*P* = 0.047) and CRF (*P* = 0.005) and negative correlation between miR‐34c and CRF (*P* = 0.038) were observed in T2DM patients when data were stratified according to the FPG level.

**Table 4:** Spearman correlation among the miRNAs and stress hormone and HOMA‐IR

	Cortisol	CRF	IL‐6	HOMA‐IR
Total subjects				
miR‐18a	0.324**	0.302**	0.177*	0.427**
miR‐34c	−0.249**	−0.266**	−0.119*	−0.416**
T2DM group				
miR‐18a	0.184*	0.257**	0.085	0.218*
miR‐34c	0.074	−0.192*	−0.158*	−0.187*
IFG group				
miR‐18a	0.182	0.289*	0.157	0.116
miR‐34c	−0.134	−0.132	−0.008	−0.125
Control group				
miR‐18a	0.100	0.026	0.081	0.088
miR‐34c	−0.041	−0.054	0.081	0.107

*P < 0.05, **P < 0.01.

CRF: corticotropin‐releasing factor, IL‐6: interleukin‐ 6, HOMA‐IR: homoeostasis model assessment of insulin.

We also found that plasma cortisol was positively related to CRF (*r* = 0.320, *P* < 0.001) and IL‐6 (*r* = 0.203, *P* < 0.001) in the study subjects. However, no significant correlation between CRH and IL‐6 was observed (*P* > 0.05).

### Relationship between miRNA expression and HOMA‐IR

As IR is a direct risk factor of T2DM, relationships between HOMA‐IR and expression levels of miR‐18a and miR‐34c were further analysed. The spearman correlation analysis showed that miR‐18a was significant positively related to HOMA‐IR while miR‐34c was negatively related to HOMA‐IR in all subjects and in subjects with T2DM separately (Table 4). To identify the relationship between miRNA and IR independent of obesity, multiple linear regression analysis was used with possible confounders, including age, smoking, drinking, physical activity, blood lipids and blood pressure were adjusted. As the results indicated, miR‐18a was a significant positive predictor of HOMA‐IR (β coefficient = 0.208; *P* < 0.001) and miR‐34c was a significant negative predictor of HOMA‐IR (β coefficient = −0.090; *P* < 0.001) independent of including WC (Table 5). If WC was replaced by BMI among the above independent variables in the linear regression analysis, miR‐18a and miR‐34c were also independently associated with HOMA‐IR (Table 5).

**Table 5:** Stepwise multiple linear regression analysis of the relationship between miRNA and HOMA‐IR

Variables	Adjusted for WC		Adjusted for BMI	
	β coefficient	*P*	β coefficient	*P*
WC/BMI	0.041	0.007	0.093	0.028
HDLC	−0.986	0.010	−1.083	0.004
miR−18a	0.208	<0.001	0.209	<0.001
miR−34c	−0.090	<0.001	−0.089	<0.001

Variables entered in step 1: age, gender, smoking, drink, exercise, TCH, TG, HDLC, LDLC, SBP, DBP, WC/BMI, miR‐18a and miR‐34c.

WC: waist circumference, BMI: body mass index, HDLC, high‐density lipoprotein cholesterol.

### The diagnostic accuracy of the miRNAs

**Figure 2:**
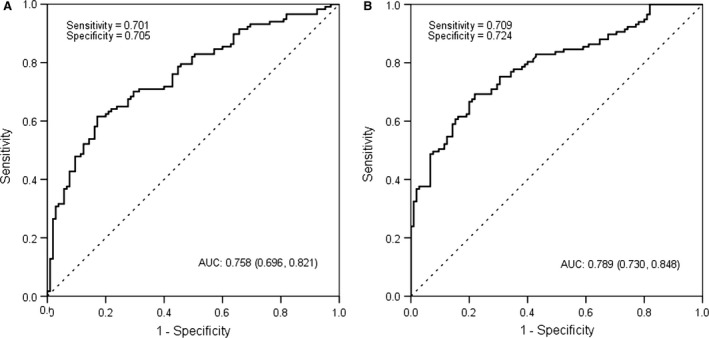
Receiver operating characteristic (ROC) curve analysis for T2DM diagnosis. Area under the curve (AUC) estimation for the microRNAs: (A) miR‐18a, (B) miR‐34c.

**Figure 3:**
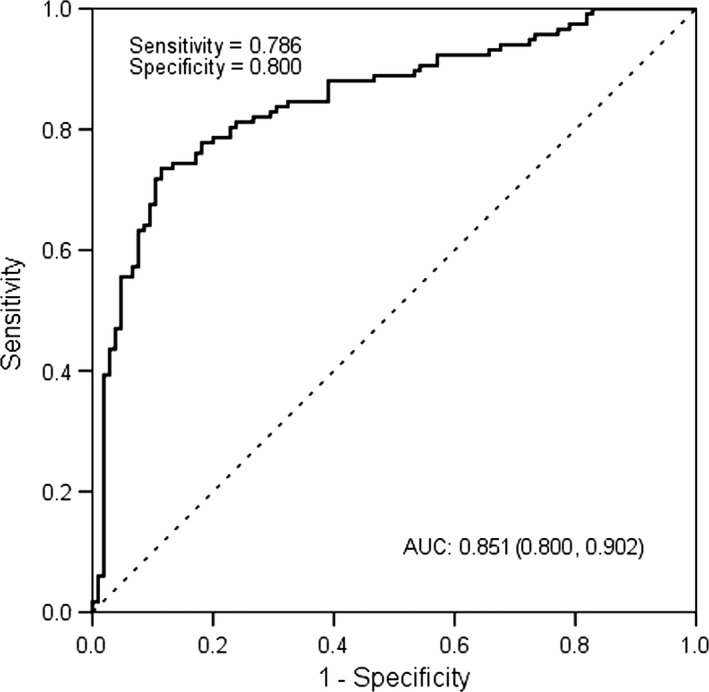
ROC plot for the microRNA panel (miR‐18a, miR‐34c) discriminating T2DM.

The diagnostic accuracy of miR‐18a and miR‐34c, measured by AUC, was 0.758 (cut‐off value: 4.72), 0.789 (cut‐off value: 11.71), respectively (*P* < 0.001). The corresponding sensitivity and specificity were presented in Figure 2. A stepwise logistic regression model to estimate the risk of being diagnosed with T2DM was applied. The two miRNAs turned out to be significant predictors (*P* < 0.001). The predicted probability of being diagnosed with T2DM from the logit model based on the two‐miRNA panel, logit (*P* = T2DM) = 0.400 + 0.374* miR18a–0.177*miR‐34c was used to construct the ROC curve. The AUC for the established miRNA panel was 0.851 (95% CI: 0.786–0.800, Fig. 3). These results reveal that miR‐18a and miR‐34c are valuable biomarkers for differentiating T2DM from healthy controls.

## Discussion

The present study examined the relationship between expression levels of two stress‐related miRNAs in PBMCs and T2DM as well as IR in an occupational sample in Beijing. We found that the average expression levels of miR‐18a and miR‐34c were significantly different between T2DM patients and healthy controls. The increased levels of miR‐18a and decreased levels of miR‐34c were associated with T2DM presence. Further, they were significant predictors for IR independent of other metabolic parameters including obesity. Our study also revealed that miR‐18a and miR‐34c in PBMCs were potential markers for diagnosing T2DM.

Growing evidence indicates that psychosocial factors play some roles in the etiology and progression of central obesity and T2DM. The possible pathophysiological mechanisms for the association between chronic psychological stress and diabetes involve hyper‐stimulation of the HPA axis which may impair glucose uptake in peripheral tissues and influence glucose metabolism by stimulating gluconeogenesis 13. Stressor exposure activates cells of the paraventricular nucleus of the hypothalamus to produce CRF, the major coordinator of the stress response. CRF acts on the anterior pituitary to promote the secretion of adrenocorticotropic hormone (ACTH), which in turn acts on the inner adrenal to initiate the synthesis and release of glucocorticoid (GC) hormones (cortisol in humans) 14. Our study showed that plasma CRF levels were significantly higher in IFG and T2DM groups than those in control group. Plasma cortisol levels were significantly higher in T2DM patients than those in IFG individuals, while the latter group showed higher levels of cortisol than the control group. These results may confirm the association between chronic stress and T2DM in an occupational sample.

The correlation between plasma levels of IL‐6 and cortisol (*r* = 0.203, *P* < 0.001) in the present study indicated the association between stress and inflammation. Many studies revealed that various psychological stressors alone can induce proinflammatory (IL‐1, IL‐6, TNF‐α) 15, 16, yet the mechanism and target cell for such cytokine production are not known for certain.

Cellular responsiveness to GCs depends on the amount of the GR protein. A recent study discovered that miR‐18 and miR‐124a might control GR activity by reducing GR protein levels in neuronal tissues 17. In correspondence, the activation of the GR‐responsive gene glucocorticoid‐induced leucine zipper was strongly impaired by miR‐18 and ‐124a overexpression. MiR‐18 is expressed widely throughout the body, while expression of miR‐124a is restricted to the brain. In small cell lung cancer, reduced GR levels have been associated with GC resistance, which might be caused by miR‐18 up‐regulation 18, 19. We found that expression levels of miR‐18a in PBMCs were positively correlated with cortisol, CRH and IL‐6. Previous investigation in occupational population showed that chronic stress increased plasma cortisol, which subsequently decreased mRNA expression of GRα and increased GRβ/GRα mRNA ratio 9. These results indicate that chronic stress up‐regulates the miR‐18a levels, which in turn reduces GR mRNA expression. Increased levels of miRNA‐18a were found to be significantly associated with T2DM, IFG and HOMA‐IR, which indicates that miRNA‐18a might be a potential marker for individuals at high risk of T2DM.

In addition, the miR‐18a is expressed as part of the OncomiR‐1 cluster, including miR‐17, miR‐18a, miR‐19a, miR‐20a, miR‐19b‐1 and miR‐92. This cluster of miRNAs was reported as potential oncogenes in various tumours. However, in our previous analysis of miRNA expression profiles (Agilent Human miRNA array V19.0), we did not find other miRNAs in the same cluster of OncomiR‐1 to be elevated in T2DM patients with increased miR‐18a.

MiR‐34c modulates cell responsiveness to CRF *via* interaction with CRFR1, which is the key upstream factor in HPA axis 8. Li's study showed that after foot shock, stressed rats showed increased levels of miR‐34c in the short‐term which trigged lower expression of CRFR1 in the hypothalamus to fight against the anxiety. After a protracted struggle which did not work, the levels of miR‐34c expression returned to normal gradually, while the levels of CRFR1 expression were up‐regulated 20. Long‐term effects of stress were demonstrated by an recent study that early life stress induced persistent increasing of central CRFR1 expression (in the hypothalamus, amygdala and the prefrontal cortex) and may explain the dysregulation of the HPA axis and increased sensitivity to stress in adult male rats 21. The negative associations between miR‐34c and CRF and cortisol in the present study might be the results of chronic effect of stress. Our finding that increased levels of miR‐34c were associated with decreased risk of T2DM, IFG and IR provides new insight that miR‐34c can alter susceptibility to stress‐related diseases by regulating gene expression. MiR‐34c up‐regulation was also found to be associated with the suppression on cell proliferation, migration and invasion of breast cancer and colorectal cancer 22, 23.

As a window, PBMCs convert psychosocial stress into cellular dysfunction and finally contribute to the pathophysiology of lifestyle‐related diseases such as diabetes mellitus, cardiovascular disease and atherosclerosis 24. The spectrum of pathologies indicates that numerous metabolic factors like glucose, lipoproteids as well as various inflammatory and anti‐inflammatory mediators lead to monocyte activation in the blood circulation 25. Up to now, most of the stress‐related miRNAs were observed based on experimental study of stressed‐rat models. It is the first time that we investigated the relationship between the two miRNAs in PBMCs and T2DM at population level. The reasonable diagnostic accuracy of miR‐18a and miR‐34c indicates their clinical value in diagnosis of T2DM. However, well‐designed prospective studies with larger sample sizes are required to validate our findings. In addition, we also recruited patients with gestational diabetes and diabetic complications in the present study. However, at the end of this study, we only recruited three individuals with gestational diabetes, eight with diabetic nephropathy and seven with diabetic retinopathy. For either miR‐18a or miR‐34c, no significant difference has been observed for the three groups when compared to the control group (*P* > 0.05). To draw a reliable conclusion based on reasonable sample size, we are still recruiting individuals with gestational diabetes and diabetic complications for further analysis.

## Conclusion

Our findings suggest that miR‐18a and miR‐34c in PBMCs may be important markers of stress reaction and may play a role in vulnerability to IR and T2DM. The miRNA panel with the two miRNAs demonstrated high accuracy in the diagnosis of T2DM. These findings may unveil new targets for the prevention, diagnosis and treatment of stress‐related disorders including T2DM.

## Conflict of interest

There was no conflict of interest in this study.
